# Review of Piezoelectric Properties and Power Output of PVDF and Copolymer-Based Piezoelectric Nanogenerators

**DOI:** 10.3390/nano13243170

**Published:** 2023-12-18

**Authors:** Neelesh Bhadwal, Ridha Ben Mrad, Kamran Behdinan

**Affiliations:** Department of Mechanical and Industrial Engineering, University of Toronto, Toronto, ON M5S 3G8, Canada; neelesh.bhadwal@mail.utoronto.ca (N.B.); behdinan@mie.utoronto.ca (K.B.)

**Keywords:** PVDF nanostructures, piezoelectric nanogenerators, piezoelectric energy harvesting, piezoelectric thin films, power output, nanoscale piezoelectric, nanocomposite, structural design, energy density, lead-free piezoelectric

## Abstract

The highest energy conversion efficiencies are typically shown by lead-containing piezoelectric materials, but the harmful environmental impacts of lead and its toxicity limit future use. At the bulk scale, lead-based piezoelectric materials have significantly higher piezoelectric properties when compared to lead-free piezoelectric materials. However, at the nanoscale, the piezoelectric properties of lead-free piezoelectric material can be significantly larger than the bulk scale. The piezoelectric properties of Poly(vinylidene fluoride) (PVDF) and Poly(vinylidene fluoride-co-trifluoroethylene) (PVDF-TrFE) lead-free piezoelectric nanomaterials are reviewed and their suitability for use in piezoelectric nanogenerators (PENGs) is determined. The impact of different PVDF/PVDF-TrFE composite structures on power output is explained. Strategies to improve the power output are given. Overall, this review finds that PVDF/PVDF-TrFE can have significantly increased piezoelectric properties at the nanoscale. However, these values are still lower than lead-free ceramics at the nanoscale. If the sole goal in developing a lead-free PENG is to maximize output power, lead-free ceramics at the nanoscale should be considered. However, lead-free ceramics are brittle, and thus encapsulation of lead-free ceramics in PVDF is a way to increase the flexibility of these PENGs. PVDF/PVDF-TrFE offers the advantage of being nontoxic and biocompatible, which is useful for many applications.

## 1. Introduction

With the increased push towards clean sustainable forms of energy, methods to convert ambient energy into useable electricity are being developed such as piezoelectric, thermoelectric, and triboelectric energy harvesters [1,2]. Due to the way dipoles are arranged in piezoelectric materials, they can transform mechanical energy (vibrations) into electrical energy and thus offer a way to harvest ambient mechanical energy. When a piezoelectric material is subjected to a force, the material deforms, causing the unit cells to deform, creating or enhancing the dipole moment in the unit cell due to the arrangement of atoms in the material. The material then has a net dipole moment, which induces charges on the electrodes of the material. This is the direct piezoelectric effect and can be used for energy harvesting. The choice between different methods of energy harvesting depends on the application. Piezoelectric energy harvesters offer the advantage of being durable and sensitive to mechanical vibrations. Lead-free piezoelectric materials are environmentally friendly when compared to chemical batteries and can supply clean renewable electrical energy [3]. A lead-free piezoelectric energy harvester could power biomedical implants, structural health-monitoring sensors, and electronics in space.

To enable the use of piezoelectric energy harvesters in more practical applications, their power density must be increased as current harvesters can only power ultra-low-power-consuming devices [4]. These harvesters should use materials with high piezoelectric properties in composite designs that improve the overall power output. The appropriateness of PVDF and PVDF-TrFE lead-free piezoelectric nanomaterials as the main piezoelectric material in piezoelectric energy harvesters is examined. The appropriateness is based on the nanomaterial’s piezoelectric properties and the power output of different PVDF/PVDF-TrFE composite structures. While numerous review papers have examined PVDF/PVDF-TrFE energy harvesters, this review is the first to systematically analyze various composite structures of PVDF/PVDF-TrFE PENGs, with a specific focus on their power output capabilities. [5,6,7].

The highest energy conversion efficiencies are typically shown by lead-containing piezoelectric materials. The harmful environmental impacts of lead and its toxicity will limit lead-based materials in future applications [3]. Several lead-free piezoelectric materials are being developed for use in energy harvesters such as zinc oxide (ZnO), aluminum nitride (AIN), barium titanate (BaTiO_3_), polyacrylonitrile (PAN), and polyvinylidene fluoride (PVDF) [3,5,8,9]. At the bulk scale, lead-free piezoelectric materials have significantly lower piezoelectric properties when compared to lead-based piezoelectric materials (can be an order of magnitude). However, at the nanoscale, the piezoelectric properties of lead-free piezoelectric materials can be significantly more substantial compared to the bulk level, primarily attributed to the heightened influence of surfaces, interfaces, decreased defects, stress concentrations, material disparities, unconfined volume expansion/contraction, elevated crystallinity, and precise management of crystal growth orientation [5,10,11,12,13]. Advances in the field of nanotechnology have paved the way for the creation of lead-free piezoelectric nanostructures undergoing further development to enhance their energy conversion efficiency [3,5,10,11,14]. Composites made from these piezoelectric nanostructures are called piezoelectric nanogenerators (PENGs) if used for energy harvesting. Piezoelectric nanogenerators (PENGs) exhibit greater flexibility compared to their larger counterparts and hold promise for electrical energy-harvesting applications. This review article will concentrate on PVDF- and PVDF-TrFE-based nanostructures and PENGs.

**Figure 1:**
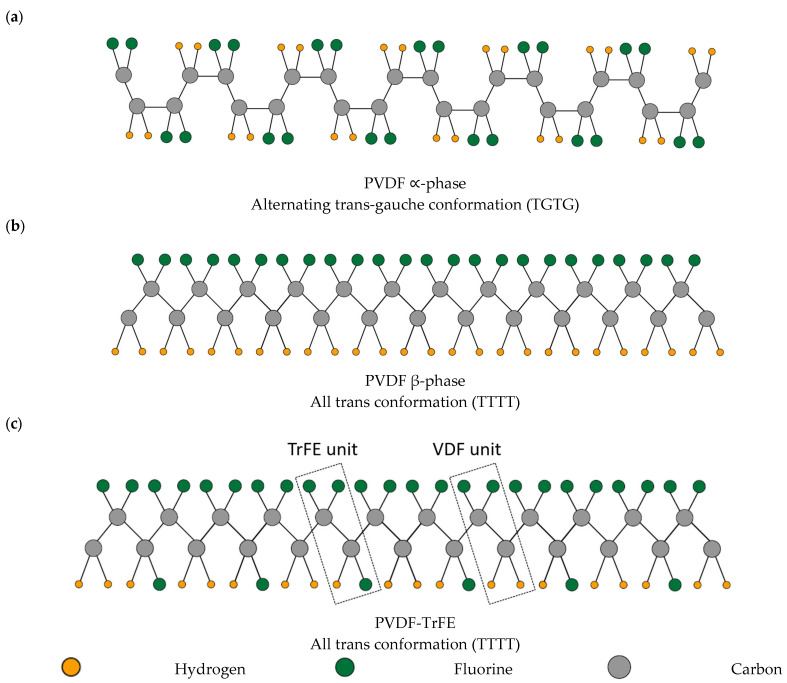
Structures of PVDF and PVDF-TrFE. (**a**) Structure of PVDF ∝-phase; (**b**) structure of PVDF β-phase; (**c**) structure of PVDF-TrFE.

PVDF is a piezoelectric polymer (chemical repeating formula (C_2_H_2_F_2_)*_n_*) discovered by Dr. Henji Kawai around 1970 in Japan [15,16,17,18,19]. PVDF is flexible [20], highly acid resistant [21], thermoplastic [22], biocompatible [22], nontoxic [22], and can be transparent under some conditions [10]. PVDF exhibits five phases, namely α, β, γ, δ, and ε, out of which the β, γ, and δ-phases show piezoelectric behavior [15,17,23,24]. The β-phase exhibits the most significant electric dipole moment, thus leading to the highest piezoelectric characteristics among all the phases [15,25]. Hence, the piezoelectric attributes of PVDF are contingent on the material’s crystallinity level and the relative composition of its various phases. The piezoelectric effect in PVDF can be limited due to its semicrystalline nature [26] and bulk PVDF is typically ~50% crystalline [27]. The α-phase has an alternating trans-gauche (TGTG) chain conformation, which leads to a mutual cancellation of dipole moments between the C–H and C–F bonds and hence the α-phase is non-polar (see Figure 1a) [15]. The β-phase has an all-trans (TTTT) conformation, where the H and F atoms are attached at both ends of the C–C chain such that the dipole moments of the two C–H and two C–F bonds add up perpendicular to the c-axis of the polymer, giving it piezoelectric properties (see Figure 1b) [15,17].

Poly (vinylidene fluoride-co-trifluoroethylene) (PVDF-TrFE) is a copolymer of PVDF and has been synthesized to achieve a higher amount of β-phase. The addition of TrFE (CF2–CFH) into VDF (CH2–CF2) promotes the immediate formation of the ferroelectric β-phase through crystallization [27,28,29,30]. In the PVDF all-trans state, the proximity of two large adjacent fluorine atoms disrupts the stability of the β-phase, leading to a preference for the α-phase with a TGTG conformation due to limited steric hindrance between the smaller hydrogen atoms and the neighboring fluorine atoms [26]. In PVDF-TrFE, the VDF and TrFE units are distributed without a specific pattern along the molecular chain and the introduction of a slightly larger fluorine atom, replacing a hydrogen atom, causes steric hindrance with neighboring G-bonds, leading to a preference for the trans bond over the gauche bond (see Figure 1c) [28]. When incorporating a minimum of 20% TrFE units, enough HH (CF2–CF2) and TT (CH2-CH2) defects are introduced, leading to significant steric hindrance within an α-phase crystal, which is not observed in a β-phase crystal, causing the copolymer chains to favor a polar all-trans conformation closely resembling the β-phase of PVDF [31]. The PVDF-TrFE copolymer with 20–50% TrFE content is more stable in the all-trans phases [26].

Table 1 lists the bulk piezoelectric properties of PVDF and PVDF-TrFE. The piezoelectric strain constant, d_33_, is the ratio of the strain produced to the applied electric field (m/V), or the ratio of electric charge generated per unit area to an applied stress (C/N) [32]. Ε_r_ is the relative dielectric constant and ε_0_ is the permittivity of free space. The piezoelectric voltage constant, g_33_, is the ratio of the strain produced to the applied electric displacement, or the ratio of the electric field generated per unit stress applied [33]. The dg product (d_33_g_33_, which is equivalent to d_33_^2^/(ε_r_ε_0_)) is a performance metric utilized for assessing the energy-harvesting potential of a piezoelectric material [33,34]. As can be seen from Table 1, PVDF-TrFE has a higher energy-harvesting capability than PVDF. For rows in Table 1 that have ranges for piezoelectric properties, values were selected to maximize the calculated d_33_^2^/(ε_r_ε_0_) in the second-last column, i.e., the lowest relative dielectric constant and highest d_33_.

Bulk PVDF and PVDF-TrFE both have negative d_33_ coefficients, as opposed to most traditional piezoelectric ceramics, which have positive d_33_ coefficients [46,47,48,49]. Physically, this means that when an electric field is exerted in the direction of polarization of a traditional piezoelectric ceramic, it will expand, while PVDF and PVDF-TrFE contract [46].

To enable the use of piezoelectric energy harvesters in more practical applications, their power density must be increased as current harvesters can only power ultra-low-power-consuming devices. These harvesters should use materials with high piezoelectric properties in composite designs that improve the overall power output. The appropriateness of PVDF and PVDF-TrFE lead-free piezoelectric nanomaterials as the main piezoelectric material in piezoelectric energy harvesters is examined. The appropriateness is based on the nanomaterial’s piezoelectric properties and the power output of different PVDF/PVDF-TrFE composite structures. While numerous review papers have examined PVDF/PVDF-TrFE energy harvesters, this review is the first to systematically analyze various composite structures of PVDF/PVDF-TrFE PENGs, with a specific focus on their power output capabilities [5,6,7]. The review discusses the PVDF/PVDF-TrFE nanostructure that exhibited the largest piezoelectric properties, as well as the PVDF/PVDF-TrFE PENG that recorded the highest power output.

## 2. Power Density-Improving Techniques

To increase the piezoelectric efficiency of PVDF, several methods and techniques have been implemented:

**Stretching**: Lovinger explained that mechanically stretching PVDF at a low temperature (~90 °C) causes the nonpolar α-phase spherulites to break and forces the molecular chains into their most extended conformation, which is the β-phase [15]. Although stretching induces the β-phase, the dipole vectors at this stage are randomly positioned within the plane perpendicular to the molecular chains [26]. Aligning the dipole vectors in the same direction is required to make the overall PVDF material demonstrate piezoelectric behavior, and this is usually accomplished by poling the material.

**Poling**: Subjecting PVDF to a strong electric field triggers the transition from the α-phase to the β-phase and aligns dipole moments along the applied electric field [15,50,51,52]. Gupta and Doughty speculated that the electrostatic force applied during the poling process can cause compression in the PVDF, leading to a relaxation of the C–C–C bond angle and an increase in the C–C separation, allowing the fluorine atoms to overcome steric hindrance, which permits the C–F bonds to rotate and facilitate the formation of the β-phase [52].

**Quenching:** Quenching PVDF at low temperatures can prompt the formation and arrangement of the self-aligned β-phase [53]. The O-H bonds in the water can form hydrogen bonds with the C–F groups of PVDF, leading to their orientation and the formation of the β-phase [53,54,55]. At lower quenching temperatures, crystallization occurs gradually, starting at the material’s surface and advancing inward through PVDF, which, in turn, results in the alignment of the β-phase during the stepwise crystallization process [53,54,55,56].

**Annealing:** Under the correct annealing process, thermal energy can promote the rearrangement of the polymer chains, inducing β-phase transformation and increasing the degree of crystallinity [57,58,59].

**Press and Fold (Hot Pressing):** High pressure and temperature can promote the α to β-phase transformation [60,61]. PVDF can be pressed and folded under high temperatures and during the process, spherulites are converted to small granular structures [60]. The β-phase has a smaller unit cell volume in comparison to the α-phase [15]. The pressure results in a closer packing of the atoms, resulting in the β-phase being preferred [60]. Temperatures between 100 and 165 °C are preferred as PVDF films prepared at temperatures below 80 °C showed obvious cracks [60].

**Electrospinning:** This technique combines both electric poling and stretching to increase the degree of crystallinity, β-phase, and dipole orientation in PVDF [62,63,64,65]. PVDF is typically dissolved in a solvent and then placed into a syringe with a metal tip. A grounded metal collector is placed in front of the syringe tip (typically 10–25 cm) and a large voltage is applied to the syringe tip, leading to a charged solution. When the electric charge force in the solution surpasses the surface tension force, a stream of PVDF dissolved in the solution is expelled from the nozzle [66]. During this process, the solvent starts evaporating, and the PVDF is poled and stretched due to the high electric field.

**Copolymers and Terpolymers:** As mentioned in Section 1, PVDF-TrFE is a copolymer that has an immediate formation of the ferroelectric β-phase through crystallization [27,28,29]. An example of a terpolymer is P(VDF-TrFE-CTFE), which is synthesized through the random addition of the third monomer chlorotrifluoroethylene (CTFE).

**Addition of Fillers:** The addition of fillers can also increase the power density of PVDF through the following mechanisms [10]: 

*β-phase increase*—The filler particles, depending on their composition, may interact electrostatically with the fluorine atoms or hydrogen atoms in PVDF, leading to the F atoms in PVDF being aligned towards or away from the filler, inducing the formation of the β-phase. Thus, the filler can act as a nucleation agent, resulting in a higher crystallinity and β-phase [10].

*Piezoelectric materials*—If the filler itself is a piezoelectric material with higher piezoelectric properties than PVDF, the filler can contribute to increasing the power output of the nanocomposite.

*Conductive materials*—Conductive fillers have the capacity to establish electrical connections within the insulating piezoelectric material, helping induced piezoelectric charges flow between the inside of the PVDF and the electrode [10,67]. This can increase power density. However, too much conductive filler can electrically connect the top and bottom electrodes, causing charge neutralization and lower output [67].

*Stress Concentrations*—Hard fillers can act as stress concentrations when subject to external forces, leading to the development of higher piezoelectric potentials [10]. The local strain in the vicinity of each filler exhibits a significantly greater magnitude compared to the bulk strain observed in pure PVDF/PVDF-TrFE film, which induces higher potential [68].

The addition of too many fillers leads to the agglomeration of nanoparticles, which can cause interfacial defects, poor mechanical properties, and overall lower electromechanical properties of the composite [69].

Often, many of these techniques are used in combination to increase the amount of β-phase, degree of crystallinity, and orientation of dipole moments in PVDF. The generation of voltage is not solely attributed to the creation of the polar β-phase. To attain PENGs with robust ferroelectric characteristics, two key requirements are essential: (i) the presence of a net dipole moment within the crystal structure and (ii) the uniform alignment of dipoles throughout the bulk material [70,71]. If the dipole moments of the β-phase are not mutually aligned, then the piezoelectric effect will be weak or nonexistent.

## 3. Piezoelectric Nanogenerator Composite Structure and Power Output

The upcoming sections delve into the power output, piezoelectric attributes, and configuration of various PVDF/PVDF-TrFE PENG composites. The classification of piezoelectric composites is determined by the number of dimensions through which the material maintains continuity, such as 1-3 or 0-3 structures, among others [72]. Traditionally, the initial digit is used to denote the phase that exhibits piezoelectric properties, and in the context of this article, it specifically pertains to PVDF/PVDF-TrFE. There can be multiple active phases.

### 3.1. (1-3 Composites) Vertically Aligned PVDF/PVDF-TrFE Nanowires and Nanotubes

**Figure 2:**
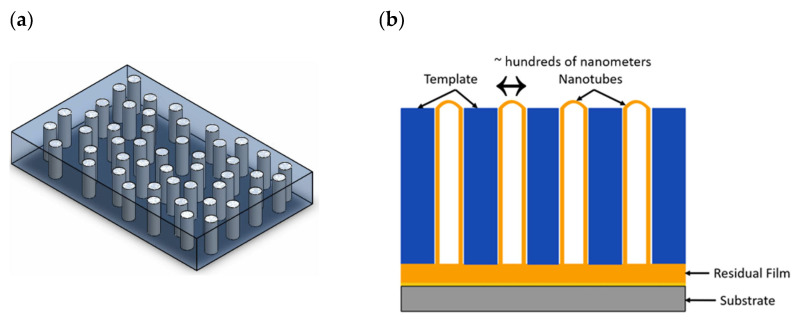

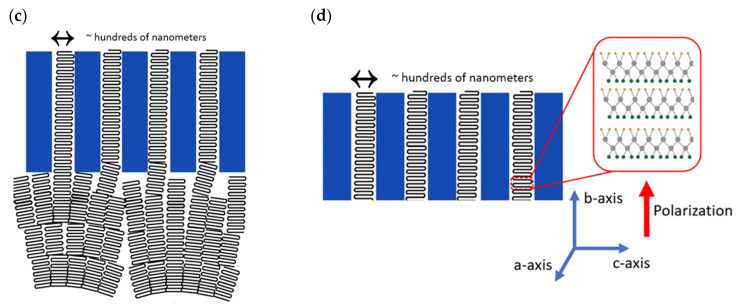
(**a**) A 1-3 PVDF/PVDF-TrFE composite with vertically aligned PVDF/PVDF-TrFE nanowires (white) encapsulated in matrix (translucent blue-grey). (**b**) Cross-section of vertically aligned nanotubes grown by Template Assisted Method. (**c**) Nanoconfinement effect: Lamellae are aligned when in the nanopores but randomly aligned in the residual film. (**d**) Orientation of PVDF chains inside nanopores; the direction of polarization is along the length of the nanopore.

PVDF/PVDF-TrFE 1-3 composites have vertically aligned PVDF/PVDF-TrFE nanotubes (NTs) or nanowires (NWs) which are uninterrupted in a single direction, while another material encapsulates the nanowires or nanotubes in all three directions as shown in Figure 2a,b. The second material may demonstrate piezoelectric activity but does not need to for example air.

It is preferred to produce this composite in a way that the dipole moments of PVDF/PVDF-TrFE are oriented perpendicular to the substrate. The b-axis (axis in the direction of polarization [15,73]) of PVDF should be perpendicular to the substrate, while the c-axis (axis along the chain direction) should be parallel to the substrate.

The most common methods used for fabricating 1-3 PVDF/PVDF-TrFE composites are as follows:

*Template-assisted method (TAM)*—In this method, a nanoporous template (usually a ceramic filter paper) is infiltrated by PVDF/PVDF-TrFE in a melt or dissolved in a solvent [74]. The polymer extends along the walls of the template, creating a thin film initially, primarily due to the fact that the forces promoting cohesion for complete filling are considerably less compared to the adhesive forces [75]. Due to nucleation and growth on the pore surface, nanotubes are formed in the case of brief infiltration periods or limited available material (see Figure 2b). Nanowires are formed due to the full filling of the template pore [74,75]. This method suffers from NW/NT entanglement and leaning issues when the template is removed.

*Nanoimprint lithography (NIL)*—In this method, a mold (typically etched silicon) with the inverse features of the PVDF/PVDF-TrFE nanowires is pressed onto a PVDF/PVDF-TrFE thin film at a temperature higher than the glass transition and Curie temperature but lower than the melting temperature of the polymer [74]. Through hot pressing, the softened polymer material is extruded to occupy the nanocavities in the mold [74]. Once cooled down, the mold is removed and the vertically aligned PVDF/PVDF-TrFE nanorods are exposed.

**Nanoconfinement:**

Nanoconfinement is an important effect that is prominent in 1-3 nanocomposites of PVDF/PVDF-TrFE where the crystallization of PVDF/PVDF-TrFE in nanopores can lead to crystal alignment with the polar b-axis of the polymer running parallel to the length of the nanopore, resulting in large piezoelectric coefficients [76].

During crystallization, heterogeneous nucleation occurs in the residual film (PVDF/PVDF-TrFE film that has not infiltrated the template) as shown in Figure 2c [77,78]. Polymer lamellae in the residual film grow radially outward from the spherulites in all directions [78]. When the lamellae hit the nanopores of the template, only lamellae with a growth direction parallel to the length of the nanopore continue to grow while the lamellae with any other growth direction get blocked by the nanopore walls and cannot continue to grow [78]. PVDF/PVDF-TrFE recrystallized without a residual film has a random orientation of polymer chains in the nanopores and hence the residual film is essential for alignment [78]. In summary, if a residual film connects the nanostructures, the dominant growth direction of the crystals (polar b-axis) is aligned along the length of the nanopores as shown in Figure 2d [77].

Table 2 compares the performance of several 1-3 PVDF/PVDF-TrFE composites. In the columns labeled power density, loading, resistor, Xc, and % β-phase are the electrical power produced by the PENG per unit area, loading conditions on the PENG during the power measurement, the value of the external resistor connected to the PENG during power measurements, degree of crystallinity, and percentage of % β-phase in the total composite (including amorphous regions), respectively. The power output of the PENGs is compared based on power density divided by the product of loading force and frequency to account for operating conditions [79]. Table 3, Table 4 and Table 5 also follow this convention. From Table 2, several 1-3 PENGs have a dg product larger than bulk PVDF and PVDF-TrFE. The 1-3 composites show high power outputs and piezoelectric coefficients because of the good mutual alignment of dipole moments due to nanoconfinement. If air encapsulates the NWs/NTs, the PENG becomes porous and there is increased stress on the NTs/NWs as the volume of piezoelectric material is reduced compared to bulk film [80].

**Nanotubes (NTs)**

In one study, a 1-3 composite with PVDF-TrFE NTs in an Anodized Alumina Membrane (AAM) was fabricated [78]. PVDF-TrFE was spin coated onto a porous anodized alumina template with one side closed (not porous) and the sample was heated to 250 °C. The polymer melt adhered to the template and entered the nanopores due to capillary forces, and this deposition process was repeated 15 times. A gold layer was deposited on the PVDF-TrFE and the bottom of the AAM template was exposed, after which the samples were poled under 800 V. There was space between the outer wall of the PVDF-TrFE nanotubes and AAM walls, which was important in not restricting the movement of nanotubes in the membrane. The piezoelectric strain coefficient of the PVDF-TrFE nanotube array was 1.97 times that of conventional spin-coated film due to several factors, including the orientation of the PVDF-TrFE crystals with their polar b-axis perpendicular to the sample surface, the removal of the substrate constraint, and the relatively low dielectric constant of the nanotube array. Bhavanasi et al. synthesized vertically aligned PVDF-TrFE nanotubes [81]. The dipoles were oriented at a 30° inclination to the length of the nanotubes, with a 40% reduction in poling field, and had a 2.2 times larger d_33_ coefficient value compared to poled films and the composite exhibited 36 times higher power output than PVDF-TrFE film due to better-oriented crystal structures together with a reduction in structural defects within nanostructures formed through the process of nanoconfinement within the template pores.

**Nanowires (NWs)**

In one study, a 1-3 composite made of PVDF-TrFE nanopillars with a diameter <20 nm and aspect ratio up to 8.9 was created [82]. A silicon mold (NIL method) was used to create two 1-3 composite films. The films were flip-stacked on top of each other such that the PVDF-TrFE pillars were touching (the electrodes were facing opposite directions) and then poled. The maximum piezoelectric strain constant of a single PVDF-TrFE nanopillar was 210.4 pm/V, while the average was 72.7 pm/V as some nanopillars did not have contact in between them and only obtained partial polarization. The average strain constant of the developed PVDF-TrFE 1-3 composite structures was 5.19 times larger than that of the PVDF-TrFE flat thin film. Chen et al. created a 1-3 composite consisting of PVDF-TrFE nanowires using a modified TAM [76]. A nanoporous anodized aluminum oxide (AAO) template was placed on top of a PVDF-TrFE layer (8–9 µm thick) and 500 V DC was applied to the top of the template at 160 °C. The generated electrodynamic force made the nanowires grow and in situ poled them as well. The template was dissolved, and the NWs were encapsulated in Poly(methyl methacrylate) PMMA. The composite showed an output voltage nine times greater than spin-coated bulk film, which was attributed to the preferential alignment of the b-axis along the vertical direction as well as an increased proportion of β-phase crystallinity in the NWs. In one study, a 1-3 composite with PVDF-TrFE nanowires was fabricated using TAM with an AAO template [83]. The d_33_ of the PVDF-TrFE nanowires was 1.6 − 2 times larger than thin films due to increased crystallization and preferential orientation of β-phase crystals along the length of the nanowires.

**Impregnated and Non-Impregnated Microwires:**

In another study, a 1-3 composite with PVDF-TrFE nanowires was fabricated using a NIL method and electrohydrodynamic (EHD) pulling [80]. PVDF-TrFE film was pressed against a PDMS mold for 30 min under a pressure of 4.8 MPa at 160 °C to form micropillars. Then, an ITO plate was placed above the pillars with an air clearance maintained with Kapton spacers and used as an upper electrode. A voltage was applied, which pulled the micropillars electrohydrodynamically (EHD) upwards, generating an array in contact with the upper electrode. The composite was annealed for 30 mins. The vertically aligned micropillars produced an output 5.4 times higher than the bulk-film-based generator, and the increase was attributed to good dipole alignment along the microwire length.

In one study, a 1-3 composite with PVDF-TrFE microwires impregnated with Boron Nitride Nanotubes (BNNTs), which are also piezoelectric, was formed using NIL [84]. PVDF-TrFE film with 0.3% weight BNNTs was hot pressed by a PDMS mold at 180 °C for 1 h to create micropillars. The mold was removed and encapsulated in PDMS. Ag nanowires were coated on the top of the PENG as the top electrode and the film was poled. The composite had a voltage output ~2.6 times that of a composite with microwires without BNNTs and an 11 times higher output than pristine flat PVDF-TrFE film. This was attributed to BNNTs acting as stress concentrations and the higher piezoelectric coefficients of BNNTs compared to PVDF and its copolymers. There were negligible changes in piezoelectric properties due to changes in the crystalline structure. The harsh conditions of a space environment require intrinsic radiation shielding and it was found that the macroscopic absorption cross-section (radiation shielding) increased by approximately 2.6 times in the 0.3 wt% BNNT micropillars when compared to pristine PVDF-TrFE. In another study, a 1-3 composite comprised of PVDF-TrFE micropillars with (20 wt%) BaTiO_3_ nanoparticles embedded in the micropillars was formed via NIL [85]. The PENG was annealed at 140 °C to obtain high crystallinity and encapsulated in PDMS. Carbon nanotubes (CNTs) were used as a top electrode (because of their exceptional electrical characteristics, strong chemical stability, and remarkable mechanical strength and flexibility). The composite was poled and provided a voltage output that was 7.3 times higher than pure PVDF-TrFE and 2.75 times higher than PVDF-TrFE micropillars without the BaTiO_3_ NPs. The superior performance of the composite was due to the higher piezoelectric properties of BaTiO_3_ and BaTiO_3_ acting as a stress concentration in the micropillars. The PENG showed good stability after 12,000 loading cycles.

**Table 2:** Comparison of 1-3 composites of PVDF/PVDF-TrFE (OC, SC, OD, ID, Ø, and l stand for open circuit, short circuit, outer diameter, inner diameter, diameter, and length, respectively. Numbers in blue are values for pure PVDF/PVDF-TrFE films/structures the author of the study made).

	Material	Poled	V (V)	I (μA)	Resistor (MΩ)	Power Density (μW/cm^2^)	Xc	% β-Phase	d_33_ (pC/N)	ε_r_	d_33_^2^/(ε_r_ε_0_) or d_33_g_33_ (m^2^/N × 10^−12^)	Power Density /(Force × Hz) µW/ (cm^2^∙N∙Hz)	Loading
[78]	PVDF-TrFE NTs (OD Ø 350 nm ID Ø 230 nm)	✓	-	-	-	-	-	-	−35 −17.8	7.7 13.2	18 2.72	-	-
[81]	PVDF-TrFE NTs (OD Ø 200 nm ID Ø 40–60 nm)	✓	4.8	-	0.576	2.2	-	-	40–44 18–24	-	-	0.37	0.075 MPa 1 Hz
[82]	PVDF-TrFE (Ø 19 nm l = 169 nm)	✓	-	-	-	-	-	-	210/ 72.7/ 14.0	-	-	-	-
[76]	PVDF-TrFE (Ø ~400 nm l = ~10 µm)	✓	1.1	-	-	-	57.7 50.8	48.2 38.2	-	-	-	-	2.5 N 2 Hz
[83]	PVDF-TrFE NWs (Ø 60 nm)	X	-	-	-	-	-	-	25–45 16–23	-	-	-	-
[80]	PVDF-TrFE Microwire (Ø ~ 8 µm l = ~50 μm)	✓	4 @10 MOhms	2.6 @ 50 kPa	2.3	5	-	-	-	-	-	0.16	30 N 1 Hz
[84]	PVDF-TrFE Microwire (Ø 80 μm l = 120 μm) MWBNNTs Ø 4.3–9.3 nm	✓	22	-	6	11.3	-	-	14 ~3.5	-	-	0.14	Compression 40 N (0.4 MPa) 2 Hz
[85]	PVDF-TrFE (Ø 22 μm l = 50 μm) BaTiO_3_ NP Ø 200 nm	✓	13.2 OC	0.33 SC	3.8	12.7	-	-	35.3 14.6	24 12	5.89 2.01	0.25	Compression 50 N (0.5 MPa) 1 Hz

### 3.2. (3-1 Composites) Piezoelectric Vertically Aligned Nanorods Encapsulated in PVDF

**Figure 3:**
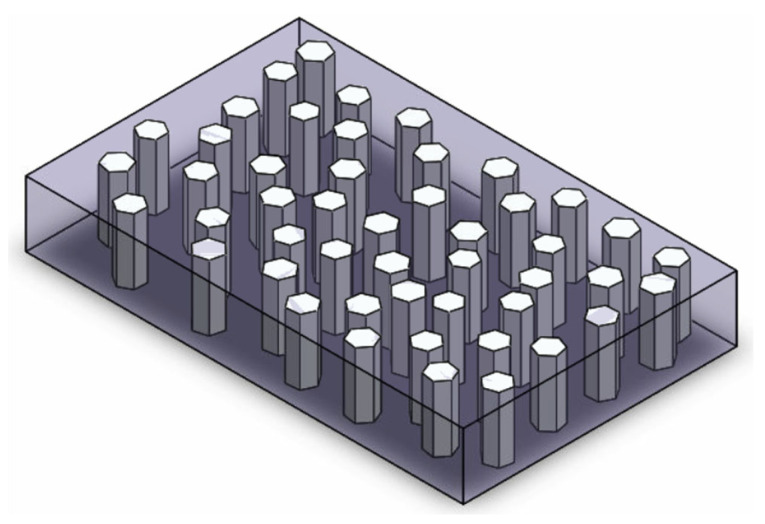
A 3-1 composite with vertically aligned ZnO NWs (white pillars with hexagonal cross-section) encapsulated in PVDF (translucent purple grey).

A 3-1 PVDF nanocomposite has vertically aligned piezoelectric nanorods (the piezoelectric material is not PVDF/PVDF-TrFE) continuous in one direction, which are encapsulated by PVDF/PVDF-TrFE in all three directions (see Figure 3). The PVDF/PVDF-TrFE encapsulant can additively contribute to the piezoelectricity of the piezoelectric vertically aligned nanorods.

Most research has been conducted on vertically aligned ZnO NRs encapsulated in PVDF to form a nanocomposite. It is preferred to fabricate this type of composite such that the c-axis of the ZnO crystals is normal to the substrate [4]. Vertically aligned ZnO nanorods are typically grown via the hydrothermal method on a substrate and then encapsulated in PVDF.

Table 3 compares the performance of several 3-1 PVDF/PVDF-TrFE composites. The 3-1 composites showed the highest power output of all the composite structures compared in this review (power density divided by the product of loading force and frequency) because of the good mutual alignment of dipole moments of the vertically aligned piezoelectric nanorods (not PVDF/PVDF-TrFE) and the PVDF/PVDF-TrFE encapsulant synergistically contributing to improving the overall power output of the nanocomposite. The vertically aligned nanorods promote the nucleation and formation of the β-phase, which increases the amount of β-phase in the PVDF. The ZnO nanorods can also act as stress concentrations.

Anand and Bhatnagar encapsulated vertically aligned ZnO nanorods in PVDF [86]. PVDF dissolved in DMF was drop cast on top of vertically aligned ZnO nanorods to form the PENG. The PENG, without any prior poling, generated a power output that was 1800 times greater than that of PVDF alone. The vertically aligned ZnO nanorods played a crucial role in promoting the nucleation and formation of the β-phase within the PVDF, and the PVDF passivated the surface of ZnO nanowires. Choi et al. synthesized vertically aligned ZnO nanowires encapsulated in PVDF [87]. ZnO NWs were encapsulated in PVDF and the tops of the ZnO nanowires were completely covered in PVDF. The voltage output was 2.7 times that of pure PVDF. Experiments showed that the ZnO NWs may not have contributed significantly to surface charge generation, but the ZnO NWs increased the strain near PVDF, which was thought to be the reason for the power enhancement. In one study, an array of ZnO was encapsulated in PVDF [88]. ZnO was grown on a PET–ITO substrate via seed layer deposition and hydrothermal growth. PVDF was dissolved in DMF and spin coated onto the substrate, after which the composite was immediately quenched at −20 °C in a solution of glycerol and water (in order to obtain β-phase). The PENG had a d_33_ value ~2.8 times higher than pure PVDF that was quenched due to a synergistic effect of ZnO NRs and PVDF when combined.

**Table 3:** Comparison of 3-1 composites of PVDF/PVDF-TrFE (OC, SC, Ø, and l stand for open circuit, short circuit, diameter, and length, respectively. No X_c_ data were given and so the column was removed. Numbers in blue are values for pure PVDF films the author of the study made).

	Material	Poled	V (V)	I (μA)	Resistor (MΩ)	Power Density (μW/cm^2^)	% β-Phase	d_33_ (pC/N)	ε_r_	d_33_^2^/(ε_r_ε_0_) or d_33_g_33_ (m^2^/N × 10^−12^)	Power Density /(Force × Hz) µW/(cm^2^∙N∙Hz)	Loading
[86]	PVDF-ZnO (Ø ~ 200 nm)	X	46.64	1.392 SC	15	45.87 1800×	94.4% of total crystallinity 53	-	-	-	3.7	Finger Tapping ~12–14 kPa 3 Hz
[87]	PVDF-ZnO (Ø hundreds of nm)	✓	~0.7 OC ~0.3 OC	~0.05 SC ~0.01 SC	-	-	-	-	~2.4 ~1.3	-	-	Bending 3.2% strain
[88]	PVDF- ZnO (Ø ~ 30 nm)	X	-	-	-	-	-	14.91 ± 4.39 5.35 ± 1.42	-	-	-	-
[89]	PVDF (Ø 160 nm)-ZnO (l = 1.5 µm Ø 120 nm)	X	0.356 OC ~0.216	0.456 SC 0.212	-	-	90% of total crystallinity	-	-	-	-	Compression 4 N 6 Hz
[90]	PVDF-ZnO (Ø 200 nm l = 3 µm)	X	2.73	152.2	0.018	103.9	-	-	-	-	-	Compression 125 Pa

In one study, vertically aligned ZnO nanorods were encapsulated in PVDF by electrospinning PVDF onto the ZnO nanorods [89]. Electrospinning was used so that post-poling treatment of the PVDF was not needed. The voltage output of the PENG was 1.6 times larger than pristine PVDF. The increase in performance was attributed to the formation of β-phase due to electrospinning, ZnO acting as a nucleating agent to induce β-phase PVDF, and the piezoelectricity of the PVDF and ZnO NRs adding constructively.

Nour et al. made two 3-1 composites of ZnO nanorods encapsulated in PVDF, which were then stacked on top of each other (face to face) [90]. Both samples were then encapsulated in PVDF via spin coating. Then, they were joined together face to face with their ZnO NWs’ top tips touching each other. The PENG had a voltage output ~1.6 times larger than the PENG without PVDF, showing the positive synergistic effect of PVDF.

### 3.3. (3-0 Composites) PVDF/PVDF-TrFE Composites with Non-Vertically Aligned Nanoparticles

**Figure 4:**
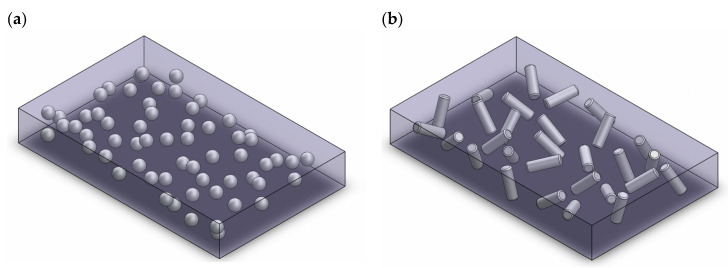
(**a**) A 3-0 composite with NPs (grey spheres) encapsulated in PVDF (translucent purple grey). (**b**) A 3-0 composite with NRs (grey rods) encapsulated in PVDF (translucent purple-grey).

In simple terms, 3-0 PVDF/PVDF-TrFE nanocomposites are PVDF/PVDF-TrFE films with nanoparticles embedded in them. The nanoparticles are non-continuous in all directions and are encapsulated in PVDF/PVDF-TrFE, which is continuous in all three directions as shown in Figure 4.

To fabricate this kind of composite, PVDF/PVDF-TrFE is typically dissolved into a solvent and then nanoparticle fillers are added to the solution. The solution is subsequently applied to a substrate using either spin coating or drop coating, after which electrodes are positioned, resulting in the creation of a 3-0 composite.

The effect of the addition of nanoparticles into a PVDF/PVDF-TrFE matrix has been discussed in Section 2 and as mentioned before, it is important that the NPs are uniformly dispersed and do not agglomerate. The main types of nanoparticles added are

1. Conductive NPs
2. Non-conductive NPs
3. Piezoelectric NPs
4. Hollow pores (pores are created in the PVDF/PVDF-TrFE film)

Methods like mechanical stretching, melt quenching, annealing at various temperatures and pressures, electrospinning, and electric poling used to enhance the β-phase, dipole orientation, and crystallinity can inadvertently introduce undesired structural deformations or microstructural defects [91]. The formation of 3-0 nanocomposites via solvent casting can be a better way to increase the β-phase and degree of crystallinity in the PVDF/PVDF-TrFE matrix [91]. However, as mentioned in Section 2, the generation of voltage is not solely attributed to the creation of the polar β-phase and to achieve PENGs with high ferroelectric properties, an identical orientation of dipoles in the bulk material is needed [70,71] Typically, studies on 3-0 composites focus on increasing the β-phase but seldom make sure that the β-phase has an identical orientation of dipoles in the bulk material.

Table 4 compares several 3-0 PVDF/PVDF-TrFE composites. A 3-0 composite is easier to manufacture compared to a 3-1 composite but exhibits reduced power outputs as seen in Table 4 due to the dipole moments of PVDF/PVDF-TrFE being randomly aligned.

**Graphene and Derivatives**

Since graphene is non-polar and PVDF is polar, the formation of homogenous composites is difficult; also, graphene is conducting, which can lead to significant dielectric loss [92,93,94]. The surface functionalization of graphene can improve dispersion. Graphene oxide (GO) has oxygen functional groups that improve dispersion by interacting with polymer chains in PVDF but it was observed that the addition of GO can lead to a deterioration in the electrical and mechanical properties of the polymer composite. Hence, Pusty et al. reduced GO to rGO to enhance dispersion in the PVDF [92]. They incorporated 1% by weight of reduced graphene oxide–silver (rGO–Ag) into the composite. The addition of Ag nanoparticles, which were easy to synthesize, was observed to improve the dielectric properties of PVDF. rGO–Ag enhanced the β and γ phases in PVDF due to the electrostatic interactions. The positively charged Ag ions were attracted to the –CF2– dipoles of PVDF, while they were repelled by the –CH2– dipoles. The PENG’s open circuit voltage and short circuit current increased by 180 and 35 times, respectively, compared to pure PVDF without poling. In another study, rGO nanosheets and bismuth aluminate (Bi_2_Al_4_O_9_) nanorods were added to a PVDF matrix [70]. Bismuth aluminate is a lead-free piezoelectric material (d_33_~−28 pC/N) but is brittle, which restricts its application. rGO nanosheets were synthesized with Bi_2_Al_4_O_9_ nanorods and the Bi_2_Al_4_O_9_/rGO nanostructures had (Bi_2_Al_4_O_9_) nanorods agglomerated with rGO nanosheets. The PVDF/ Bi_2_Al_4_O_9_/RGO composite had a significantly higher power density, approximately 20 times greater than that of pure PVDF. This was attributed to (i) the piezoelectric properties of the Bi_2_Al_4_O_9_ nanorods, (ii) the presence of RGO nanosheets that create a conductive path facilitating charge movement toward the electrode surfaces, and (iii) a higher proportion of the β-phase in PVDF due to interactions between the polar surface of Bi_2_Al_4_O_9_ and RGO nanofillers with the PVDF.

**ZnO**

In one study, a PVDF film with nanopores was synthesized [68]. ZnO nanoparticles (50 wt%) were added to a solution of N, N-DMF, and PVDF. The solution was drop cast to form a film, which was annealed and then etched in HCl to remove the ZnO nanoparticles and form a porous PVDF film. The film was then poled. The PENG had an output current ~11 times higher compared to a pure PVDF-based PENG. ZnO was used to create porosity and to promote the formation of the β-phase through the dipolar interaction. The pores increased the stress in the PENG and boosted piezo potential. In one study, ZnO nanoparticles were added to PVDF to increase the β-phase [95]. The negatively charged surface of the ZnO NPs increased the β-phase and the degree of crystallinity by ~2 times. The combination of the piezoelectric effect due to PVDF and ZnO NPs leads to a higher piezoelectric output. Singh et al. added ZnO NRs (15 wt%) to PVDF to increase the β-phase of PVDF [96]. The degree of crystallinity and percentage of β-phase increased due to electrostatic interactions between ZnO and PVDF. The PENG had an increased open-circuit voltage by ~6 times compared to pure PVDF and the author attributed the increase to β-phase formation, and the contribution from the piezoelectric properties of ZnO was insignificant in this context because the ZnO particles were randomly oriented within the composite films.

**Other Materials**

Numerous studies have concentrated on increasing the β-phase in PVDF by incorporating nanoparticles (NPs). Karan et al. added 5 wt% vitamin B2 (VB2) powder to PVDF to create a completely organic-based biocompatible PENG [97]. VB2 contains hydroxyl, carbonyl, and amino groups that effectively stabilize the polar β-phase of PVDF through hydrogen bonding, leading to an increase in crystallinity and β-phase content. The PENG had an output voltage and current 26 times and 40 times higher, respectively, when compared to pure PVDF. Electrical poling resulted in only a 1.04 times increase in output voltage, suggesting that the PENG was self-poled. The device showed durability and stability in performance for 10 weeks. Panda et al. added 8 wt% of an n-type semiconductor material, calcium titanate (CTO) perovskite powder, to PVDF [98]. The addition of CTO increased the β-phase and power output of the film due to the interaction between CTO particles and PVDF resulting in an output voltage around four times higher than pure PVDF. The biocompatibility of the film was tested via NIH3T3 cells and biocompatibility was indicated. In one study, TiO_2_ nanoparticles were added to PVDF [91]. TiO_2_ has high chemical stability, thermal stability, and high dipole moment. The addition of TiO_2_ significantly increased the β-phase and piezoelectric properties of PVDF due to dipole–dipole interactions between the nanofiller and the matrix. The degree of crystallization, fraction of β-phase, and output voltage increased with the addition of 10 wt% TiO_2_ to the PVDF matrix. In one study, 10 wt% BaTiO_3_ (BTO) NPs were added to PVDF [99]. The dielectric constant roughly doubled at 1 kHz compared to pure PVDF. BTO is lead-free but brittle, and this can be resolved by adding BTO NPs to PVDF, further increasing the β-phase and internal polarization of the PVDF.

Wang et al. added 10% piezoelectric EDABCO-CuCl_4_ (EDABCO = N-ethyl-1,4-diazoniabicyclo[2.2.2] octonium) nanoparticles to a PVDF matrix [79]. In contrast to pure PVDF, the introduction of EDABCO-CuCl_4_ nanoparticles resulted in an enhancement, with a two-fold increase in output voltage (OC), a three-fold increase in current density (SC), and a remarkable twenty-eight-fold increase in output power. The output voltage of the nanocomposite was tested before and after poling, and poling did not affect the output performance.

**Table 4:** Comparison of 0-3 composites (OC, SC, Ø, and l stand for open circuit, short circuit, diameter, and length, respectively. Numbers in blue are values for pure PVDF/PVDF-TrFE films the author of the study made).

	Material	Poled	V (V)	I (μA)	Resistor (MΩ)	Power Density (μW/cm^2^)	X_c_	% β-Phase	d_33_ (pC/N)	ε_r_	d_33_^2^/ε_r_ε_0_ or d_33_g_33_ (m^2^/N × 10^−12^)	Power Density /(Force × Hz) µW/(cm^2^∙N∙Hz)	Loading
[92]	PVDF- rGO Ag	X	18 OC 0.1 OC ×180	1.05 SC 0.03 SC ×35	1	0.36	46 9	β ~17% γ~14%	-	-	-	0.012	Hand Tapping 4.6 kPa ~5 Hz
[70]	PVDF/ Bi_2_Al_4_O_9_ (l = 100 nm) rGO	X	5.92 OC 1.34 ×4.41	0.76 SC 0.22 ×3.45	12	0.457	-	-	-	-	-	3.5	Finger tapping 10–12 kPa ~2 Hz
[68]	Porous PVDF (Pore Ø 60 nm)	✓	84.5 OC 10.5 ×8	22 SC 2 ×11	7	12	-	-	-	-	-	0.18	2 kPa 30 Hz
[95]	PVDF- ZnO NR Ø ~ 50–150 nm	X	24.5 OC 4.8 ×5.1	1.7 SC 0.41 ×4.1	-	-	64.1 ~30	53.84	50.4 ~22.3	~22 ~8	13 ~7.0	-	Finger motion 28 N 5 Hz
[96]	PVDF-ZnO NR	X	1.81 OC ~0.3 ×6	0.56 SC ~0.34 ×1.6	7	0.21	55.36 28.98	42	−1.17	-	-	3.4 × 10^−6^	~15 kPa ~2 Hz
[97]	PVDF- VB2	X	61.5 OC 2.3 ×26.7	12.2 SC 0.3 ×40.6	8	300.5	~53 39	β~51% γ~2%	−50.3 −40.7	~53 ~9	5.41 20.8	1.3	~80 N ~3 Hz strain rate 0.797% s^−1^ 500 kPa
[98]	PVDF-CaTiO_3_	✓	20 ×4	0.25 ×2	100	0.19	-	-	- 1540	~18	-	0.24	~5 N ~0.16 Hz
[91]	PVDF-TiO_2_ NP <100 nm	X	5.45 OC 2.08 ×2.6	-	-	-	38.22 26.78	34.01	-	23.06 @ 1 × 10^6^ Hz	-	-	Finger tapping ~4 Hz
[99]	PVDF-BaTiO_3_ (~460 nm)	✓	7.2	0.038	100	0.8	-	-	-	47 ~23	-	-	10 m/s^2^ 1.68 Hz
[79]	PVDF-EDABCO-CuCl_4_ (50–150 nm)	X	63 OC ×2	2.1 SC ×3	8	43.7	-	-	-	5.3	-	0.58	Compression 50 kPa 15 N 5 Hz

### 3.4. Electrospun Fibers

The process of electrospinning has already been described in Section 2. Electrospinning as a fabrication method of PVDF/PVDF-TrFE PENGs is considered to be a relatively simple low cost operation [6]. The piezoelectric properties of electrospun PVDF/PVDF-TrFE fibers are increased due to the mechanical stretching and poling of the fibers, and PENGs prepared by this method typically do not require a post-poling process as used in other fabrication methods.

**Figure 5:**
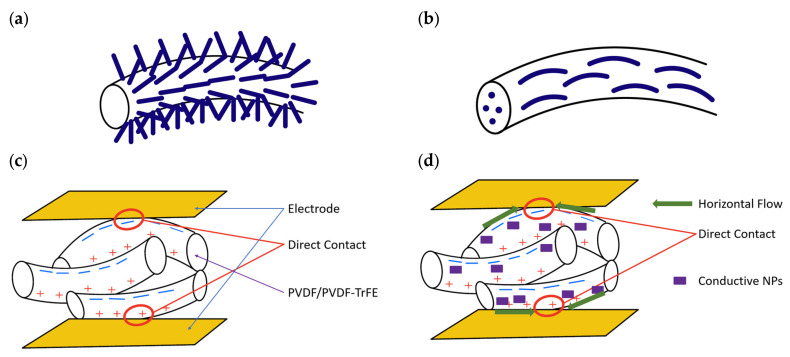

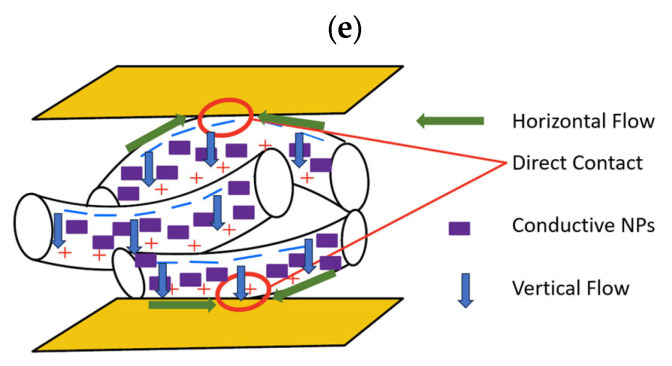
(**a**) ZnO NWs grown on the surface of a PVDF/PVDF-TrFE fiber. (**b**) NPs in a piezoelectric fiber of PVDF/PVDF-TrFE. (**c**) Only charges making direct contact with the electrode of an electrospun fiber are used. (**d**) Conductive NPs increase the horizontal flow and more charges can be used. (**e**) Addition of too many conductive NPs increases volume conductivity and can short the PENG.

The fabrication of this type of PENG typically involves the addition of PVDF/PVDF-TrFE into a polar solvent to form a solution. NPs may be added to the solution, which is then electrospun. The advantages of adding NPs to the PVDF/PVDF-TrFE matrix have been explained in Section 2 and the final structure is shown in Figure 5a,b. Several parameters that affect the quality and output of electrospun PENGs are humidity, temperature, applied electric field, solvent type, solution concentration, feed rate, collector type, distance between the syringe nozzle and collector, and syringe nozzle opening [6,100,101].

Once the electrospun mat is created, typically electrodes are fixed on the top and bottom of the mat. The electrodes are only partially in contact with a restricted number of nanofibers on the surface layer of the PENG and as a result, the piezoelectric charges generated by nanofibers that are not in direct contact are not utilized, as shown in Figure 5c [67]. Increasing the surface conductivity of PVDF/PVDF-TrFE fibers with the aid of conductive nanoparticles facilitates the lateral movement of the generated piezoelectric charges between the fibers on the surface layer, as depicted in Figure 5d [67]. However, an excess of conductive nanoparticles in the electrospun PVDF/PVDF-TrFE PENG results in a more rapid increase in volume conductivity compared to surface conductivity, which causes the induced charges to flow longitudinally, leading to neutralization and leakage effects, ultimately reducing the piezoelectric output voltage, as illustrated in Figure 5e [67].

Table 5 compares several electrospun PVDF/PVDF-TrFE fibers. Electrospun fibers can show degrees of crystallinity higher than bulk-scale PVDF as seen in Table 1 and Table 5. This is because of poling and stretching during electrospinning that increases the degree of crystallinity, β-phase, and mutual orientation of dipole moments. The power output of electrospun fibers is less than that of 1-3 composites due to higher porosity and less direct contact with electrodes, and typically, the electrospun mats are tested under compression where the direction of force is perpendicular to the fiber length and dipole moment direction.

**Increasing Dispersion of NPs**

Several studies focused on improving the dispersion of NPs in electrospun PVDF-TrFE. Shi et al. created electrospun PVDF-TrFE with 10 wt% BaTiO_3_ nanowires (NWs) coated in PMMA via atom transfer radical polymerization to improve dispersion [69]. The NWs were well oriented along the length of the electrospun fiber. The maximum output power of the 10 wt% PMMA-coated BaTiO_3_/PVDF-TrFE PENG was 2.2 times greater than the maximum output power of the 10 wt% BaTiO_3_/PVDF-TrFE PENG (without PMMA coating), and it was also 7.6 times higher than the maximum output power of the PVDF-TrFE-based PENG. The higher output was due to the piezoelectricity of the BaTiO_3_ NWs, improved dispersion of the BaTiO_3_ NWs, and the high Young’s modulus of PMMA, which significantly enhanced the efficiency of stress transfer at the interface between the BaTiO_3_ nanowires (NWs) and the PVDF-TrFE matrix. The PMMA also reduced leakage current through the composite. The PENG was stable for 6000 cycles. In one study, PVDF was electrospun with BaTiO_3_ nanoparticles (15 wt%) and graphene nanosheets (0.15 wt%) [102]. The output voltage was ~3.8 times, ~2 times, and ~2.8 times higher than pure PVDF, BaTiO_3_-PVDF, and graphene NS-PVDF-based PENGs, respectively, and was attributed to an increase in the β-phase, piezoelectric contributions from BaTiO_3_, BaTiO_3_, and graphene acting as stress concentrations, increased uniform dispersion of BaTiO_3_ (due to the influence of graphene on the clustering or aggregation of BaTiO_3_ (BT) nanoparticles [103]), and the development of conductive networks because of the conductive graphene nanosheets (NSs), which lead to an improved transfer of induced charges. The increase in β-phase was attributed to hydrogen-bonding interactions between the fluorine (F) atoms in PVDF and the hydrogen (H) atoms in the hydroxyl groups on the surfaces of BaTiO_3_ [104]. Additionally, the hydrogen (H) atoms of PVDF were drawn towards the graphene surface because of the electrostatic interaction between the highly electronegative carbon (C) atoms in graphene and the less electronegative hydrogen (H) atoms within the PVDF chains [105,106]. While electrospinning, the presence of conductive graphene amplifies the local electric field, leading to a more potent Coulomb force attracting PVDF chains, prompting them to crystallize into the β-phase on the graphene surface. The PENG demonstrated stable output voltages over a period of 1800 cycles. Zhang et al. electro-sprayed PVDF with BiCl_3_ and ZnO NPs [107]. The output voltage and degree of crystallinity increased by approximately four times and two times due to the addition of the nanoparticles, respectively, and were attributed to an increase in the β-phase due to interfacial interactions between ZnO, BiCl_3_, and PVDF. BiCl_3_ also consumes part of the surface hydroxyl groups of ZnO nanoparticles, resulting in a more uniform dispersion of the ZnO NPs in the PVDF increasing β phase. Bairagi et al. electrospun PVDF with KNN (3 wt%) and CNTs (1 wt%) [108]. The PENG had a voltage output ~2.6 times higher than the PENG without any CNTs. The enhancement was credited to the even distribution of KNN nanorods within the PVDF, facilitated by the presence of carbon nanotube (CNT) fillers, the CNTs and KNN acting as nucleating agents, the higher amount of mechanical stretching during electrospinning due to the conductive CNT filler, and the CNT filler providing a conductive path into the nanocomposite. Both the KNN nanorods and CNT filler were aligned along the axis of the fibers, a configuration that enhances the piezoelectric effect.

**Carbon-based NPs**

Yang et al. electrospun PVDF with (2 wt%) reduced graphene oxide nanoparticles in it [109]. GO nanoparticles were mixed in N, N-dimethylacetamide (DMF) and then PVDF was added and electrospun under a voltage of 1.4 kV/cm. The PENG was dried for 2 h at 80 °C after which it was heated for 1 h at 140 °C to reduce GO to rGO. The PENG had an open circuit voltage output ~11 times higher than pure PVDF and ~3.7 times higher than PVDF–GO PENG. The increased output was attributed to increased β-phase due to the graphene, the graphene acting as stress concentrations, and the conducting network being formed due to the graphene, which improved the transfer of induced charges generated by PVDF. Both graphene oxide (GO) and reduced graphene oxide (rGO) had a positive impact on the output of the PENG. However, rGO had a more pronounced effect in improving PENG performance because rGO is more conductive than GO because rGO has fewer functional groups attached to the carbon atoms, enhancing its conductivity. In one study, PVDF with a Ce^3+^ complex (Cerium (III)-N,N-dimethylformamide-bisulfate [Ce(DMF) (HSO_4_)_3_]) along with graphene NSs was electrospun into a PENG [110]. The PENG had an open circuit voltage ~2.5 times that of the PENG without graphene NSs. The enhanced output was attributed to the conductivity of the graphene NSs, the increase in crystallinity, and an increase in the β phase due to the interactions of the fillers with PVDF. In one study, PVDF with 5 wt% MWCNTs (multi-wall carbon nanotubes) was electrospun [67] and the resulting PENG produced an output voltage three times higher than pure PVDF. Multi-walled carbon nanotubes (MWCNTs) were oriented along the fiber axis and increased the quantity of the β-phase because the PVDF chains crystallized into the β-phase while on the surface of MWCNTs during the electrospinning process. Furthermore, the MWCNTs elevated the surface conductivity of PVDF fibers, promoting the preferential lateral movement of induced piezoelectric charges between the fibers on the surface layer, as depicted in Figure 5d.

**Other NPs**

Tiwari et al. electrospun PVDF with two-dimensional nanoclay platelets, i.e., Cloisite 30B (bis-(hydroxyethyl) methyl tallow ammonium ion-exchanged montmorillonite [111]. The PENG had an output circuit voltage of 3.5 times higher than pure PVDF. The increase in performance was attributed to the increase in the β-phase due to the interaction between the nanoclay and PVDF matrix as well as the nanoclay acting as a nucleating agent. The nanoclay created a minute mesh-like structure, which, in turn, impeded the propagation of cracks, resulting in a more durable fiber when nanoclay was present. The tensile strength, Young’s modulus, and toughness of the PENG were 3.3, 3.4, and 3.5 times greater than that of pure PVDF, respectively. This enhancement in toughness and modulus was ascribed to changes in the material’s morphology, crystal structure, and the effective dispersion of the layered silicates within the polymer matrix.

In one study, pure PVDF was electrospun [66]. Several electrospinning parameters such as polymer solution preparation, applied voltage, distance to collector distance, collector type, flow rate, and electrospinning nozzle were optimized, and a PVDF PENG with a power output of 2.2 μW/cm^2^ was synthesized. Liu et al. electrospun PVDF as well as polyacrylonitrile (PAN), another piezoelectric polymer, to form a membrane [112]. PVDF and PAN powder were mixed in a glass beaker (5:3 mass ratio) after which a DMF:acetone mixture (8:2) by volume was added and mixed at 60 °C for 2 h. During electrospinning, the solution led to the crystallization of PAN into a ferroelectric phase with a planar zig-zag chain conformation. Subsequently, the dipolar interactions between the CH_2_CH≡N dipoles in PAN and the CH_2_CF_2_ dipoles in PVDF induced the formation of the β-phase. The planar zig-zag chain conformation in PAN also contributed to the piezoelectricity of the PENG. Moreover, the tensile strength of the PENG was 7 MPa and the output was stable for 4000 cycles under a compressing force.

**ZnO**

In a previous review, we reviewed ZnO NPs as an additive to electrospun PVDF/PVDF-TrFE fibers and included this in this review as well [4]. The advantages of adding NPs to the PVDF/PVDF-TrFE matrix have been explained in Section 2. Additionally, the growth of ZnO nanostructures on the surface of PVDF/PVDF-TrFE fibers, as illustrated in Figure 5a, can enhance the presence of the β-phase. This is a result of the nano forces induced on the fiber surface during the growth of ZnO nanostructures.

In one study, ZnO nanorods (27.3% by mass) were grown on top of electrospun PVDF [113]. The electrospun PVDF fibers were dip coated into a seed solution of ZnO and then ZnO was grown via hydrothermal synthesis. The PVDF–ZnO PENG produced an output voltage that was nearly three times higher compared to a pure PVDF PENG prepared using the same method. This boost in performance was attributed to the ZnO nanorods deflecting and sliding against each other during vibrations, causing greater deformation of both the ZnO nanorods and the PVDF fibers, ultimately increasing the power output. In another study, PVDF was electrospun, and ZnO nanorods were grown on top of the structure [114]. The open-circuit voltage experienced an approximately 2.3-fold increase in the presence of ZnO nanorods. Additionally, it was found that the mat possessed a level of breathability similar to cotton.

In one study, ZnO nanorods (5 wt%) were added to PVDF, which was then electrospun [115]. In this research, ZnO nanoparticles (NPs) and ZnO nanorods (NRs) were compared as fillers. The output voltage of the NR-PVDF mat was approximately 1.4 times that of the NP-PVDF mat and approximately 4 times that of the pure-PVDF PENG. The ZnO nanorods were aligned along the fiber axis due to their substantial aspect ratio. They served as β-phase nucleating agents with a good orientation brought about by electrostatic interactions, resulting in a higher β-phase content compared to ZnO nanoparticles. Furthermore, their large aspect ratios made them more easily deformable by external forces. Ye et al. electrospun PVDF-TrFE with ZnO nanorods (10 wt%) [116]. The PENG had a voltage output ~5 times that of a pure PVDF-TrFE PENG. The PENG had a higher β-phase, which was attributed to PVDF-TrFE’s interactions with ZnO NRs as well as ZnO NRs having a higher piezoelectric coefficient than PVDF-TrFE. Yi et al. electrospun PVDF with Y-doped ZnO NSs (15 wt%) [117]. The voltage output increased by around three times. The Y-doped ZnO NSs led to stress concentrations (knots) in the PVDF fibers and increased the β-phase percentage due to the interaction of the filler and PVDF. Aligned dipoles of Y-doped ZnO NSs with the fiber axis induced more dipole orientation in the PVDF. In one study, PVDF was electrospun with KNN (3% by wt) and ZnO (2% by wt) nanorods [118]. The PENG output was 2.4 times higher compared to the PENG with only PVDF and KNN. This increase in output was because of the β phase nucleating effect due to ZnO nanorods, and the challenge of hindering β-phase formation due to the presence of KNN nanorods inside the PVDF matrix was addressed by adding ZnO nanorods. Both KNN and ZnO contributed to the output because of their piezoelectric properties. Additionally, the semiconductive nature of ZnO nanorods offered an enhanced conductive pathway within the PVDF polymer matrix. Moreover, the alignment of both KNN and ZnO nanorods in a uniaxial fashion within the fiber further played a role in these improvements. In one study, PVDF-HFP (polyvinylidene fluoride hexafluoropropylene) was electrospun with co-doped ZnO (2 wt%) [119]. The PENG exhibited a voltage output that exceeded that of a pure PVDF-HPF by more than 20 times. This significant improvement was attributed to an increase in the β phase, which occurred due to the interaction between the oppositely charged Co-ZnO surface and the -CF_2_/CH_2_ dipoles of PVDF-HFP.

**Table 5:** Comparison of electrospun composites (OC, SC, Ø, and l stand for open circuit, short circuit, diameter, and length, respectively. Numbers in blue are values for pure PVDF/PVDF-TrFE electrospun fibers the author of the study made).

	Material	V (V)	I (μA)	Resistor (MΩ)	Power Density (μW/cm^2^)	X_c_	% β-Phase	d_33_ (pC/N)	ε_r_	d_33_^2^/(ε_r_ε_0_) or d_33_g_33_ (m^2^/N × 10^−12^)	Power Density /(Force × Hz) µW/(cm^2^∙N∙Hz)	Loading
[69]	PVDF TrFE (BaTiO_3_ NPs l = 2.6–3.9 µm Ø 150–300 nm)	12.6 OC	1.3 SC	7.2	4.25 ×7.6	-	94.4% of total crystallinity	-	-	-	-	Bending 2 Hz frequency 4 mm displacement
[102]	PVDF (~Ø 0.6 µm) (BaTiO_3_ NPs Ø 200 nm and Graphene NSs)	~5.5	~0.8	6.9	0.65	-	91.1% of total crystallinity	-	-	-	-	Bending (slider) 2Hz frequency 4 mm displacement
[107]	PVDF (Ø 0.37 µm) with BiCl_3_ and ZnO NPs Ø ~ 30 nm	12 OC ~3	~0.08 SC ~0.02	1000	0.64	75.54 38.76	69 15	3.8 1.74	-	-	-	0.5 Hz translation stage 6mm tensions
[108]	PVDF (Ø 163 nm)-KNN CNTs	12	18	0.220	54	-	82.5% of total crystallinity	-	-	-	2.25	Compressive 1 kPa 60 Hz
[109]	PVDF Ø 0.1~0.25 μm (reduced graphene oxide)	~8.5	-	10	3.7	-	87% of total crystallinity ~82%	-	-	-	-	Finger pressing 2 Hz
[110]	Ce^3+^ doped PVDF Ø ~ 80 nm graphene NSs	~5	~0.003 Per cm^2^	1	0.56	56 53	46% ~42%	-	-	-	0.0175	Compression 8 N 6.6 kPa 4 Hz
[67]	PVDF (average Ø 600–700 nm)-MWCNTs	6 OC 2 OC	-	-	-	38.1 47	26 0	-	-	-	-	Bending 3 cm displacement 0.8 Hz
[111]	PVDF (Ø 330 ± 30 nm)- Cloisite 30B	~15	~16	1	68.0 23.2	-	79% of total crystallinity ~66%	-	-	-	-	Finger Tapping
[66]	PVDF	0.32	-	594	2.2	-	-	41.38–18.2	-	-	0.00049	Compression 35 Hz ~128 N
[112]	PVDF/PAN Ø 100–300 nm	1.3 OC	0.07 SC	-	-	-	83.4% of total crystallinity	-	-	-	-	Compression 1 N 2 Hz
[113]	PVDF (Ø 219.4 nm)-ZnO (Ø 90–140 nm)~300 nm long	1.12	1.6	0.7	0.2	-	73.2% of total crystallinity ~70.8%	-	-	-	1.6 × 10^−5^	140 Hz 116 dB
[114]	PVDF (Ø 120 ± 100 nm)-ZnO (l = ~183 ± 153 nm Ø 30 ± 9 nm)	8.3	0.139	60	0.077	-	80.2% of total crystallinity ~83.8%	-	-	-	0.00051	0.1 MPa 1 Hz
[115]	PVDF (1.28 µm)-ZnO NR (Ø 70 nm l = 850 nm)	85 OC	2.2 SC	-	-	53.1 ~46%	48.1 ~39.6%	-	-	-	-	Bending 4 Hz
[116]	PVDF-TrFE (Ø.98 µm) -ZnO NRs Ø 91 nm l = 793 nm	61 OC	2.2 SC	-	-	63 ~44	~58 ~41	-	-	-	-	Finger bending 4 Hz
[117]	PVDF (Ø 178 nm)-Y-doped ZnO	13	1.6	10	2	-	72% of total crystallinity 69	-	-	-	0.06	40 N 0.8 Hz
[118]	PVDF (Ø 300 nm)/KNN /ZnO (Ø 79 nm)	8.31	5	10	10.38	84	78.92	-	-	-	0.43	Sewing machine 1 kPa 60 Hz
[119]	PVDF-HFP—Co-doped ZnO	2.8 OC ~0.120	-	-	-	35 21	19.11 7.87	-	38 8	-	-	Tapping force 2.5 N 50 Hz

## 4. Discussion and Conclusions

The appropriateness of PVDF/PVDF-TrFE as a lead-free piezoelectric material for use in PENGs was examined. Comparing the properties of PVDF/PVDF-TrFE in Table 1, Table 2, Table 3, Table 4 and Table 5, we find that the form of PVDF/PVDF-TrFE with the highest d_33_ coefficient was PVDF-TrFE nanowires, which had a d_33_ value of 210.4 pm/V, which is around half that of PZT in bulk form and around five times that of bulk PVDF-TrFE [82]. Good dipole alignment from nanoconfinement could be the major factor in the high d_33_ value. The form of PVDF/PVDF-TrFE with the highest degree of crystallinity was electrospun PVDF, which had a degree of crystallinity of ~75%, which is ~50% higher than bulk PVDF [107]. This was attributed to interactions between the polymer and filler as well as the electrospinning process, where the polymer was stretched and poled simultaneously. Clearly, the piezoelectric properties of PVDF/PVDF-TrFE can be significantly enhanced at the nanoscale when compared to the bulk. To attain robust ferroelectric characteristics, two key requirements are essential: (i) the presence of a net dipole moment within the crystal structure and (ii) the uniform alignment of dipoles throughout the bulk material [70,71]. In bulk PVDF, there are several phases, some of which are not piezoelectric; hence, the piezoelectric attributes of PVDF are contingent on the material’s crystallinity level and the relative composition of its various phases. The piezoelectric effect in bulk PVDF is limited due to its semicrystalline nature [26] and is typically ~50% crystalline [27]. Methods described in Section 2 and effects such as nanoconfinement can greatly boost the piezoelectric properties of PVDF/PVDF-TrFE.

**Figure 6:**
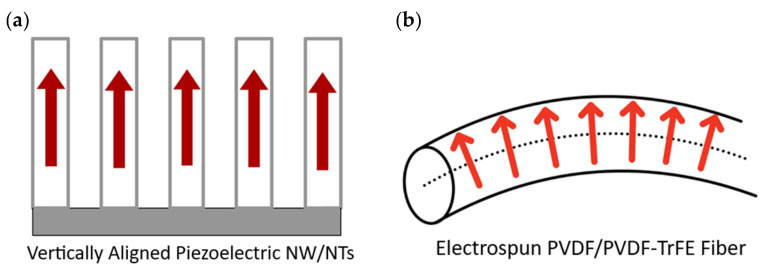

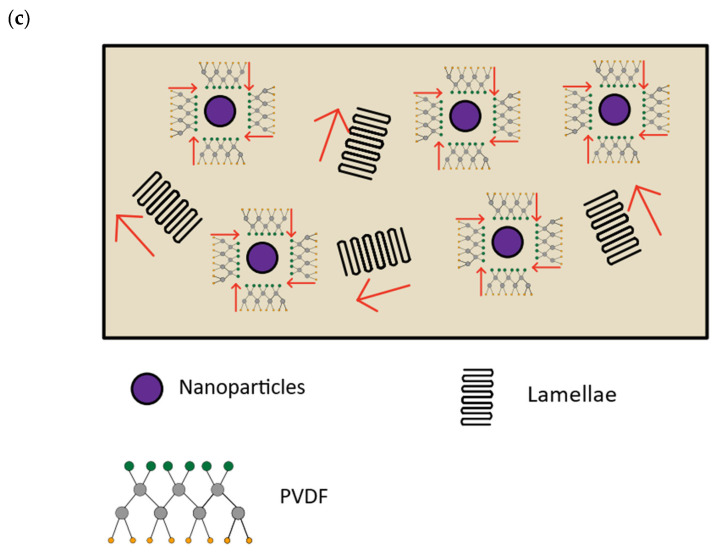
Alignment of dipoles in (**a**) 1-3 and 3-1 composites (depiction is a cross-section). The dipoles are aligned along the length of the nanowire; (**b**) electrospun fibers. The dipoles are aligned perpendicular to the length of the nanowire; (**c**) 3-0 composites (depiction is a cross-section). The dipoles are randomly aligned.

The biggest factor when determining which composite structure provides the highest power output is how identical the dipoles are in alignment throughout the composite. Figure 6 shows the direction of dipoles in different composite structures discussed in the review. Figure 6a shows that the dipoles are all vertically aligned in 1-3 PVDF/PVDF-TrFE composites due to the nanoconfinement effect. In 3-1 ZnO composites, the dipoles of ZnO are vertically aligned along the length of the nanowire like Figure 6a. In 1-3 and 3-1 composites, the dipoles are all orientated in the same direction and hence the composite has high power output. In Figure 6b, the dipoles in an electrospun fiber are perpendicular to the length of the fiber [120,121]. In this case, if multiple fibers are on top of each other, the fibers will have some bends in them, and the dipoles may start pointing in different directions. The power output of these kinds of composites is generally lower than 1-3 and 3-1 composites because of bending leading to dipole misalignment and lower density as these fibers have space in between them. Figure 6c depicts 3-0 composites with a nanoparticle filler shown in purple. The interaction of the nanoparticles with the PVDF in the depiction causes the fluorine atoms in PVDF to orient themselves towards the nanoparticle. There are also lamellae oriented in different directions as shown in the depiction. The dipole moments are oriented in different directions and can cancel each other out, leading to low power output.

Comparing Table 2, Table 3, Table 4 and Table 5, the composite structure with the highest power output divided by the product of loading force and frequency was a 3-1 composite with vertically aligned ZnO nanowires encapsulated in PVDF [86]. This was due to the good mutual alignment of dipole moments of the vertically aligned ZnO nanowires and the PVDF encapsulant synergistically improving the overall power output of the PENG. The vertically aligned nanorods promoted the nucleation and increase in the amount of β-phase in the PVDF.

Overall, PVDF and PVDF-TrFE can show significantly increased piezoelectric properties at the nanoscale compared to bulk scale but these values are still lower than lead-free ceramics at the nanoscale [4]. If the sole goal in developing a lead-free PENG is to maximize output power, lead-free ceramics at the nanoscale should be considered. However, lead-free ceramics are brittle, and thus encapsulation of lead-free ceramics in PVDF is a way to increase the flexibility of these PENGs. PVDF and PVDF/TrFE offer the advantages of being nontoxic and biocompatible, which are useful for several PENG applications.

## 5. Future Directions

Future directions for study are as follows:

1. The use of multiple techniques described in Section 2 to increase the power output of PVDF/PVDF-TrFE PENGs.
2. The complete characterization of PVDF/PVDF-TrFE PENGs in terms of frequency response and piezoelectric properties so that future PVDF/PVDF-TrFE PENGs can be designed via computational methods to resonate at the low frequencies found in ambient conditions [122,123,124,125].
3. Previous studies have shown that shear mode harvesters have higher power density compared to other modes [34,126] and thus PVDF/PVDF-TrFE PENG shear mode studies are needed.
4. A study of the long-term durability and biocompatibility of PVDF/PVDF-TrFE PENGs is needed before these PENGS can be commercially used.

## Figures and Tables

**Table 1:** PVDF and PVDF-TrFE bulk-scale piezoelectric properties.

Material	Values	Crystallinity	% β-Phase	d_33_ (pC/N)	ε_r_	d_33_^2^/(ε_r_ε_0_) or d_33_g_33_ (m^2^/N × 10^−12^)	Reference
PVDF (Bulk)	Generally Accepted Values	~50–60% [15]	-	−13–(−35)	8–15	17	[25,35,36,37,38]
	Different Industrial Datasheets	-	-	−30	8–10	12	[39]
		-	>80% of total crystallinity	−23–(−28)	13.5	6.6	[40]
		-	-	−33	-	-	[41]
PVDF-TrFE (Bulk)	Generally Accepted Values	-	-	−24–(−39)	5–20	34	[25,35,37,38,42]
	Different Industrial Datasheets	80–90% [43]	-	>25	7.5–8.5	9.5	[44]
		-	-	−38	7.9	21	[45]
